# Emergent neuropsychiatric symptoms as preclinical flags of cognitive decline: Clinical implications

**DOI:** 10.1002/alz.70849

**Published:** 2025-10-28

**Authors:** Min Tang, Mengting Yang, Yongzhi Xie

**Affiliations:** ^1^ Department of Geriatrics The Third Xiangya Hospital Central South University Changsha China; ^2^ Department of Neurology The Third Xiangya Hospital Central South University Changsha China; ^3^ Department of Radiology The Third Xiangya Hospital Central South University Changsha China

**Keywords:** Alzheimer's disease, cerebrospinal fluid biomarkers, Geriatric Depression Scale, Mild Behavioral Impairment Checklist, Neuropsychiatric Inventory

Dear Editor,

We congratulate Kim et al. for an elegant longitudinal study linking the late‐life emergence of clinically meaningful neuropsychiatric symptoms (NPS) to accelerate cognitive decline and a > 3‐fold higher hazard of incident mild cognitive impairment (MCI), with convergent evidence from time‐varying Cox models and mixed‐effects trajectories.^1^ From a clinical perspective, several points may help contextualize and extend the authors’ findings.

First, the observation that new, clinically significant NPS in cognitively unimpaired (CU) older adults presage MCI aligns with recent evidence: a 2024 systematic review and meta‐analysis found that NPS in non‐demented populations significantly increase risk of MCI/dementia.^2^ This strengthens the case for routine NPS surveillance as part of preclinical risk assessment, complementary to the 2024 Alzheimer's Association biologically based diagnostic and staging framework that emphasizes biomarkers while encouraging incorporation of clinical features that foreshadow symptomatic stages.^3^

Second, we endorse the authors’ decision to privilege specificity (Neuropsychiatric Inventory [NPI] ≥ 11, Mild Behavioral Impairment Checklist [MBI‐C] ≥ 7, and Geriatric Depression Scale [GDS‐15] ≥ 5) over ultra‐low thresholds that may capture transient distress. The MBI‐C cutoff around 7 has been used in recent validation studies and clinical cohorts and offers a pragmatic anchor for case finding. That said, clinical practice benefits from distinguishing *persistent* NPS (≥ 6 months; the core MBI criterion) from brief episodes, because persistence appears to carry stronger prognostic weight. When feasible, a sensitivity analysis using MBI‐consistent persistence (and domain‐level MBI‐C scores) could clarify which behavioral syndromes—apathy, affective dysregulation, impulse dyscontrol, social inappropriateness, or psychosis—most strongly drive risk.

Third, the gradient of conversion risk across the NPS × phosphorylated tau (p‐tau)181 groups is clinically compelling. Two extensions would aid translation: (1) formally test *statistical interaction* between time‐varying NPS and baseline tau (rather than only four strata) within the Cox model; and (2) repeat the analysis with *blood‐based* tau (p‐tau181 or p‐tau217), which are increasingly prognostic and far more scalable for memory clinics and primary care. Recent work shows plasma p‐tau181 enhances the prediction of near‐term clinical decline, and 2024 criteria explicitly incorporate blood‐based biomarkers into staging.^3^, ^4^

Fourth, this single‐center, highly educated cohort limits external validity; nevertheless, the clinical message is actionable. In practice, when new, clinically meaningful NPS arise after age ≈ 60, we suggest: screen with a brief NPS tool (MBI‐C or NPI‐Q) plus a mood scale (e.g., GDS‐15), exclude reversible contributors (pain, sleep apnea, polypharmacy, substance use, bereavement), document a cognitive baseline and arrange follow‐up within 6 to 12 months, and in patients with persistent NPS or added risk factors (apolipoprotein E ε4, family history), consider blood‐based biomarkers (p‐tau181/217) and/or referral to a memory clinic. Such a pathway may also help address the persistent under‐recognition of early cognitive impairment in primary care, where detection rates remain low.^5^

Last, because a small subset progressed CU → dementia without an intermediate MCI diagnosis, a sensitivity analysis censoring these individuals or applying interval‐censoring methods would confirm robustness. Additionally, reporting the distribution of psychotropic medication initiation around NPS onset would help clinicians interpret whether treatment masked or modified trajectories.

## 1 CLINICAL TAKEAWAY

Kim et al. provide persuasive evidence that *late‐life, clinically significant NPS are not benign*: they herald measurable cognitive decline and—especially when paired with Alzheimer's disease pathology biomarkers—identify CU adults at materially higher short‐ to mid‐term risk. Embedding structured NPS surveillance alongside evolving blood‐based biomarker strategies offers a practical route to earlier identification and risk‐adapted follow‐up in everyday care.

## AUTHOR CONTRIBUTIONS

Conceptualization: Min Tang, Yongzhi Xie; methodology: Min Tang, Mengting Yang; formal analysis: Mengting Yang; data curation: Mengting Yang; investigation: Min Tang, Mengting Yang; visualization: Mengting Yang; writing—original draft: Min Tang; writing—review & editing: Mengting Yang, Yongzhi Xie; supervision: Yongzhi Xie; project administration: Yongzhi Xie. All authors approved the final manuscript and accept responsibility for the content.

## CONFLICT OF INTEREST STATEMENT

The authors declare no conflicts of interest. Author disclosures are available in the supporting information.

## Supporting information



Supporting Information

## References

[1] Kim TH , Head E , Stark CEL , et al. Late‐life emergence of neuropsychiatric symptoms and risk of cognitive impairment in cognitively unimpaired individuals. Alzheimers Dement. 2025;21(8):e70619. 40851415 10.1002/alz.70619 PMC12375875

[2] Huszár Z , Engh MA , Pavlekovics M , et al. Risk of conversion to mild cognitive impairment or dementia among subjects with amyloid and tau pathology: a systematic review and meta‐analysis. Alzheimers Res Ther. 2024;16(1):81. 38610055 10.1186/s13195-024-01455-2 PMC11015617

[3] Jack CR, Jr , Andrews JS , Beach TG , et al. Revised criteria for diagnosis and staging of Alzheimer's disease: Alzheimer's Association Workgroup. Alzheimers Dement. 2024;20(8):5143‐5169. 38934362 10.1002/alz.13859 PMC11350039

[4] Devanarayan V , Llano DA , Hu YH , et al. Plasma pTau181 enhances the prediction of future clinical decline in amyloid‐positive mild cognitive impairment. Alzheimers Dement (Amst). 2024;16(3):e12621. 39045143 10.1002/dad2.12621 PMC11263975

[5] Liu Y , Jun H , Becker A , Wallick C , Mattke S . Detection rates of mild cognitive impairment in primary care for the United States Medicare population. J Prev Alzheimers Dis. 2024;11(1):7‐12. 38230712 10.14283/jpad.2023.131 PMC10995024

